# The Bioconcentration and Degradation of Nonylphenol and Nonylphenol Polyethoxylates by *Chlorella vulgaris*

**DOI:** 10.3390/ijms15011255

**Published:** 2014-01-17

**Authors:** Hong-Wen Sun, Hong-Wei Hu, Lei Wang, Ying Yang, Guo-Lan Huang

**Affiliations:** MOE Key Laboratory of Pollution Processes and Environmental Criteria, College of Environmental Science and Engineering, Nankai University, Tianjin 300071, China; E-Mails: hhw1070063@163.com (H.-W.H.); wang2007@nankai.edu.cn (L.W.); yangying@163.com (Y.Y.); yaotingyu@nankai.edu.cn (G.-L.H.)

**Keywords:** bioconcentration, degradation, nonylphenol, nonylphenol polyethoxylates, *Chlorella vulgaris*

## Abstract

Nonylphenol polyethoxylates (NPnEOs), a major class of nonionic surfactants, can easily enter into aquatic environments through various pathways due to their wide applications, which leads to the extensive existence of their relative stable metabolites, namely nonylphenol (NP) and mono- to tri-ethoxylates. This study investigated the bioconcentration and degradation of NP and NPnEO oligomers (*n* = 1–12) by a green algae, *Chlorella vulgaris*. Experimental results showed that *C. vulgaris* can remove NP from water phase efficiently, and bioconcentration and degradation accounted for approximately half of its loss, respectively, with a 48 h BCF (bioconcentration factor) of 2.42 × 10^3^. Moreover, *C. vulgaris* could concentrate and degrade NPnEOs, distribution profiles of the series homologues of the NPnEOs in algae and water phase were quite different from the initial homologue profile. The 48 h BCF of the NPnEO homologues increased with the length of the EO chain. Degradation extent of total NPnEOs by *C. vulgaris* was 95.7%, and only 1.1% remained in water phase, and the other 3.2% remained in the algal cells. The algae removed the NPnEOs mainly through degradation. Due to rapid degradation, concentrations of the long chain NPnEO homologous in both water (*n* ≥ 2) and the algal phase (*n* ≥ 5) was quite low at the end of a 48 h experiment.

## 1. Introduction

Nonylphenol polyethoxylates (NPnEOs) are one class of most widely used non-ionic surfactants. Internationally, NPnEOs have been used in many different industrial processes, such as laundering, textile processing (wetting and scouring), pulp and paper processing, paint and resin formulation, oil and gas recovery, steel manufacturing, pest control and power generation for over 50 years [1–3]. The wide use of products containing NPnEOs made these compounds frequently detected in the environment. The environmental fate of NPnEOs has been well documented [4–6], and about 60% of the surfactant production enters into the aquatic environment via various pathways such as municipal and industrial wastewater discharges and sewage treatment plant effluents [7]. NPnEOs are partially converted to more persistent and toxic metabolites such as nonylphenol (NP), short chain mono- to triexthoxylates (*i.e*., NP1EO, NP2EO, NP3EO) [4,8–12], and hence, these compounds frequently coexist in surface waters as the result of discharges from waste water treatment plants that are inefficient at removing nonylphenolic compounds.

Some of the degradation products, especially NP, are more lipophilic and consequently much more toxic than their parent compounds [6,13–16]. Moreover, it has been confirmed that NP and their polyethoxylates have antiandrogenic activity, for instance, disturbing the proper functioning of androgens that are essential for the normal development of males and their reproductive systems for both marine and fresh water species [17,18]. Other adverse effects were also reported. For examples, they have a significant toxic effect on hepatocytes *in vivo* [19], and on splenocytes and sertoli cells *in vitro* [14,20]. Hence, the widespread use of NPnEOs, coupled with the frequent detection of their relatively stable toxic metabolites, has led to restrictions in their production and application. The European Directive No. 2003/53/EC [21] prohibited the use of NP and its ethoxylates in the European Union. The U.S. Environmental Protection Agency (U.S. EPA) has issued a specific action plan [22] to support and encourage the ongoing voluntary phase-out of NPnEOs in industrial laundry detergents in order to manage potential risks from NPnEOs. However, the application is not prohibited globally, and relatively high concentrations of NPnEOs and NP were still found in rivers and lakes in China, Japan, USA and other countries, with concentrations varying widely from tens of nanograms per liter to more than one hundred micrograms per liter [23–30], particularly in areas affected by wastewater effluents [31–35].

The occurrence of NP and other NPnOEs biodegradation metabolites in surface waters has been studied extensively and a substantial body of research has been developed about the toxicity and bioconcentration of these compounds [36–41]. Degradation of NP, NP1EO has been studied with aerobic microorganisms and anaerobic microorganisms, respectively. Under aerobic conditions, Yuan *et al.* [42] found that *Pseudomonas* sp. expressed the best biodegrading ability among several microorganism species tested. Chang *et al.* [43] observed degradation of NP and NP1EO under different anaerobic conditions and the results showed that the degradation rates follow an order of sulfate-reducing condition > methanogenic conditions > nitrate-reducing conditions. Though with relatively high water solubility, the NPnEOs, and especially their small metabolites that have relatively greater lipophilicity, show substantial bioaccumulation factors. The estimated bioconcentration factors in fish tissues range from 13–410 for NP, 3–300 for NP1EO and 3–330 for NP2EO [44].

Although the toxic or inhibitory levels of NP and NPnEOs on algae were successfully identified in previous studies [1,13,36,45–48], little is known about the bioconcentration and biodegradation of NP and NPnEOs by microalgae. Among aquatic organisms, microalgae play an important role in the monitoring of aquatic pollution because they are at the bottom of the aquatic food chain, and the concentration of pollutants in algae may be transferred to organisms at higher trophic levels through the food chain. Moreover, algae have been shown to be capable of degrading organic pollutants including aromatic compounds, azo dyes, phthalates and tributyltin [49–51]. Thus, proliferation of an algal component may enhance the degradation process of a chemical in an aquatic environment. Only a few studies have reported the bioconcentration of NP and NPnEO in algae. Ahel *et al.* [44] reported that rather high concentrations of NP, NP1EO, and NP2EO occur in macrophytic algae, particularly in *Cladophora glomerata*, with bioconcentration factors of NP of up to 10,000. Correa-Reyes *et al.* [15] studied NP algal bioconcentration using microalgae *Isochrysis galbana*, and found that the algae was able to bioconcentrate NP by 6940 times. However, the biodegradation of NPnEO and NP by microalgae has never been studied. Therefore, the present work undertakes an investigation on the concentration and degradation of NP and NPnEOs (*n* = 1–12) by a green algae, *Chlorella vulgaris*. The bioconcentration tendency of NPnEO homologues with different chain lengths was discussed. Moreover, the contributions of bioconcentration and biodegradation to the dissipation of NPnEO in an aquatic environment were analyzed.

## 2. Results and Discussion

### 2.1. Bioconcentration and Biodegradation of NP by *C. vulgaris*

**Figure 1.**
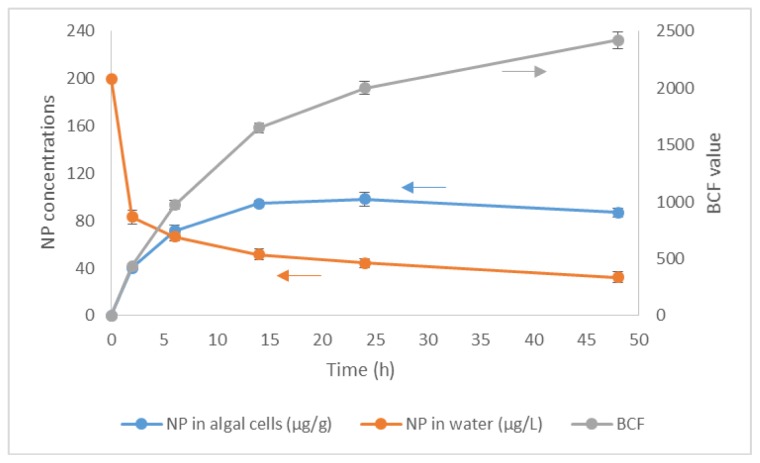
Nonylphenol (NP) concentrations in algal cells and water phase, and bioconcentration factors (BCFs) of NP in algal cells with time during incubating *C. vulgaris* in 200 μg/L NP solution.

Considerable NP was concentrated in the algal cells (Figure 1), which increased quickly with time first and stabilized or even diminished a little after a maximum was arrived. The concentrations of NP in the algae were 98.3 μg/g at 24 h (peak) and 86.9 μg/g at 48 h, respectively. Accordingly, the concentration of NP in water declined rapidly in the first 2 h to 53.7% of the initial concentration of 200 μg/L, after that, the changes of concentration of NP was not so dramatic, and NP decreased to 74.7% at 14 h and 82.1% at 48 h, respectively. Correa-Reyes *et al.* [15] also found that bioconcentration of NP in algae is a rapid process, and after *I. galbana* was incubated with 100 μg/L NP for only 1 h, most of the NP was accumulated in the algal cells and almost no free NP remained in the medium.

Bioconcentration factor (BCF) was calculated by the ratio of the concentrations in the algae and in the aqueous phase (Figure 1) [52]. BCF was 2422 at 48 h, and 2001 at 24 h. A little bigger BCF was achieved at 48 h due to the fact that both the concentrations in the algae and water phase had decreased since those at 24 h. This indicated that NP was degraded in the algae culture. Besides, the algal density increased a little during the experiment, which may also lead to the reduction of NP concentration in the algae due to the growth dilution effect.

**Figure 2.**
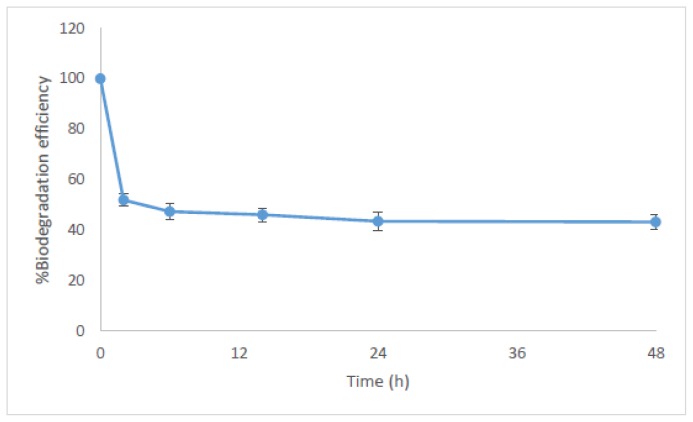
Time dependent NP biodegradation efficiency by *C. vulgaris* based on the total NP mass lost in algae and water phase.

To confirm the degradation of NP in the algal culture, the total NP mass in the culture was calculated (Figure 2). It can be seen that based on the total mass of NP in algal cells and water phase, NP degradation was rapid during the initial period. The degradation ratio was 48.0% at 2 h, and then the degradation slowed down, with degradation efficiencies of 52.6% at 6 h and 56.7% at 24 h, respectively. It should also be noted here that an unknown peak appeared on the chromatogram after NP, which could not be identified as the breakdown product of NP, and it should be an oxidative conjugate NP [53] and need further study.

The bioaccumulation of NP in aquatic organisms at different trophic levels was widely studied [36]. The freshwater algae, fish and invertebrates have been tested in the laboratory [5]. Snyder *et al.* [54] reported BCFs of 245–380 in fathead minnow. As for saltwater species, Ekelund *et al*. [55] reported a BCF of 12,500 in stickleback (*Gasterosteus aculeatus*). Correa-Reyes *et al.* [15] reported microalgae *I. galbana* was able to bioconcentrate NP by 6940 times, where 77% of the initial NP is accumulated intracellularly after 1 h of incubation, suggesting that the bioconcentration of NP in freshwater algae is considerable. Apart from the studies of bioaccumulation of NP, the degradation of NP by microorganisms has also accumulated a lot of data. Chang *et al.* [42,56,57] conducted tests on the anaerobic degradation of NP by microorganisms in sludge, river sediment and mangrove sediment. They isolated pure microbial strains from sludge samples and eight bacterial strains were capable of degrading NP anaerobically, using it as a carbon source. Gabriel *et al.* [58] described the bacterial metabolism of several NP isomers and found that the initial reaction of NP degradation was anipso-hydroxylation producing 4-alkyl-4-hydroxy-cyclohexadienones. Microorganisms and rat liver microsomes use the same type of mechanism to detach different substituents in ortho- and para-substituted phenols. In this experiment, the degradation of NP by microalgae was for the first time reported and it is obvious that *C. vulgaris* can degrade NP effectively.

### 2.2. Bioconcentration and Biodegradation of NPnEOs (n = 1–12) by *C. vulgaris*

**Figure 3.**
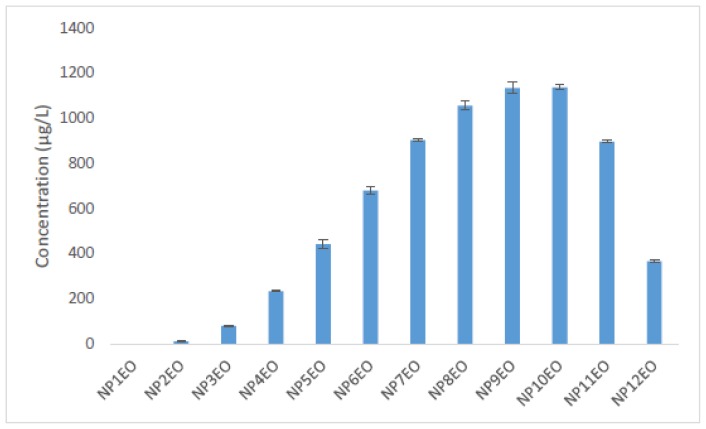
Initial concentration distribution profiles of the nonylphenol polyethoxylates (NPnEO) homologues (*n* = 1–12).

Figure 3 illustrates the initial concentration distribution profiles of all homologues of the tested NPnEOs (*n* = 1–12) in water, which is showing a Poisson distribution. The maximum concentration appears at NP9EO and NP10EO, the concentration of bilateral homologues shows a declining trend, and NP1EO was almost undetected.

**Figure 4.**
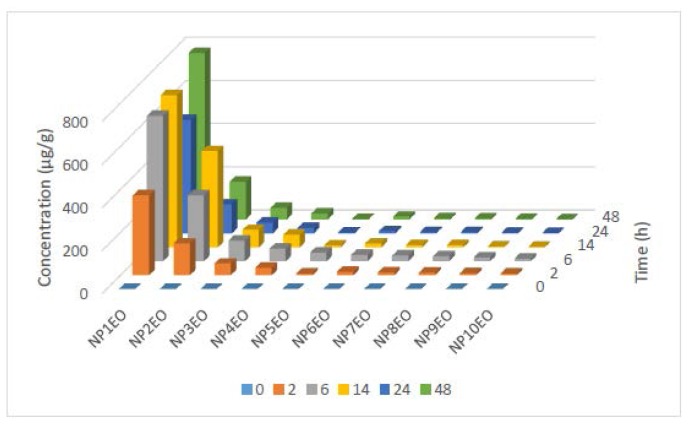
Time-dependent bioconcentration of the homologues of NPnEOs (*n* = 1–12) in *C. vulgaris*.

Figure 4 shows the concentration of all NPnEO homologues (*n* = 1–12) in the algal cells of *C. vulgaris*. NP1EO had the highest concentration in algal cells, and the enrichment reduced gradually with the increasing number of EO groups. Comparatively speaking, the enrichment of short chain NPnEOs (*n* < 5) was bigger than the longer homologues (*n* ≥ 5). The two homologues with the longest EO chain, NP11EO and NP12EO, could not be detected in the algae though their concentrations in water were substantially high (Figure 3). This is mainly due to the different lipophilicity of the different homologues. As the number of EO groups increases, the lipophilicity of the homologues decreases [59–61]. Besides, when a chemical molecule is too large, it becomes difficult for the chemical to penetrate the biomembrane. In addition, the homologues with long EO chains can be degraded more quickly than those with short chain homologues [62].

The bioconcentration kinetics of the different NPnEO homologues by *C. vulgaris* varied a lot (Figure 4). The concentration of NP1EO in *C. vulgaris* reached 372.4 μg/g at 2 h; increased gradually to 706.3 μg/g at 14 h; and at the end of 48 h experiment, reached the maximum value of 772.3 μg/g. Compared with the other long chain homologues, it is evident that NP1EO showed the greatest accumulation. Different from NP1EO that the concentration showed an increasing trend along the whole experiment course, other homologues all showed a peak value. The peak is due to the simultaneous absorbance and degradation in algal cells. NP2EO also showed a substantial concentration, with the maximum concentration of 447.4 μg/g at 14 h. The concentrations of NP3-4EO were relatively lower as compared to NP1-2EO, reaching peak values of 96.1 and 59.2 μg/g, respectively. The concentrations of longer chain homologues were further lower, and at 6 h, the concentrations reached the maximum values of 41.2 μg/g for NP5EO, 29.7 μg/g for NP6EO, 26.4 μg/g for NP7EO, 23.3 μg/g for NP8EO, 18.9 μg/g for NP9EO and 12.9 μg/g for NP10EO, respectively.

**Figure 5.**
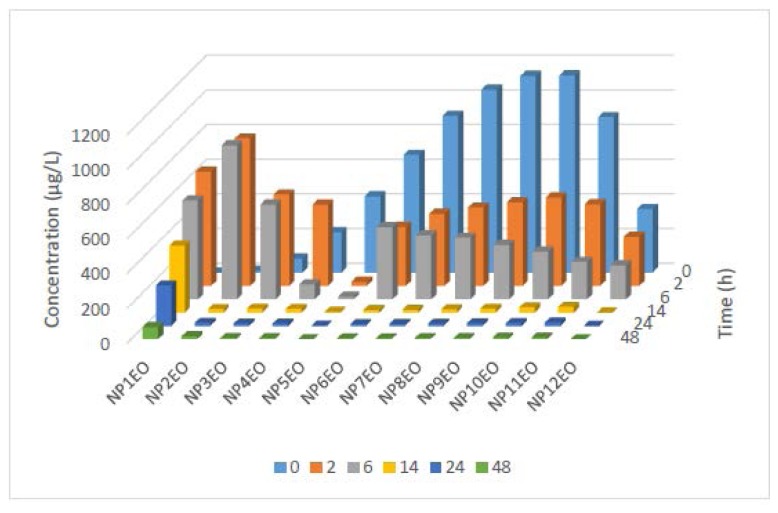
Variation of concentration of the NPnEO (*n* = 1–12) homologues in water phase.

The water phase concentration of the NPnEO homologues also followed different changing trends. The concentrations of short chain NPnEOs (*n* < 5) in water appeared an increasing trend in the initial period (2–6 h), and then decreased (Figure 5). Oppositely, the concentration of long chain NPnEOs (*n* ≥ 5) decreased constantly in the course of experiment, except that NP6EO showed a slight increase during 2–6 h. The dramatic reduction of the long chain NPnEOs could not be explained solely by the uptake by the algae, for NP11EO and NP12EO, was not detected in the algal cells. Degradation is the main reason for the reduction of long chain NPnEOs. This could be confirmed by the increase in water concentration of short chain NPnEO. The accumulation of short chain NPnEOs both in water phase and algal cells during the initial period of the experiment is due to the fact that long chain NPnEOs have greater degradation rates than the short ones. It has been recognized that NPnEOs initially break down into a series of biodegradation intermediates, including low-mole ethoxylates (retaining mainly one- and two-mole ethoxylate units), ether carboxylates (carboxylation of the ethoxylate group) [11,63]. At the end of the 48 h experiment, the concentration of all NPnEO homologues (*n* = 1–12) in water is very low, except for NP1EO and NP2EO, which demonstrates that *C. vulgaris* can remove NPnEOs (*n* = 1–12) effectively in water.

BCF values of all the NPnEO homologues in *C. vulgaris* were calculated for all the sampling times (Table 1). The time dependent trend of BCF was not the same with those of algal concentrations. This is because both algal phase concentration and water phase concentration varied intricately due to the simultaneous biodegradation and uptake of NPnEO by the algae. Generally, BCF reached a peak value at the end of experiment due to the low water phase concentration. The maximum BCF of single NPnEO homologue occurred at 14 h for NP2EO, indicating the successive degradation from long chain homologues to short chain ones inside the algal cells. The 48 h BCFs of NPnEO homologues in *C. vulgaris* ranged from 171–11,028, declining with increasing EO chain, with an exception of NP4EO. This demonstrates the remarkable discrepancy in eco-risk of different NPnEO homologues. The exception of NP4EO could be explained by the relative biodegradation rates and relative bioconcentration tendencies of different NPnEO homologues.

**Figure 6.**
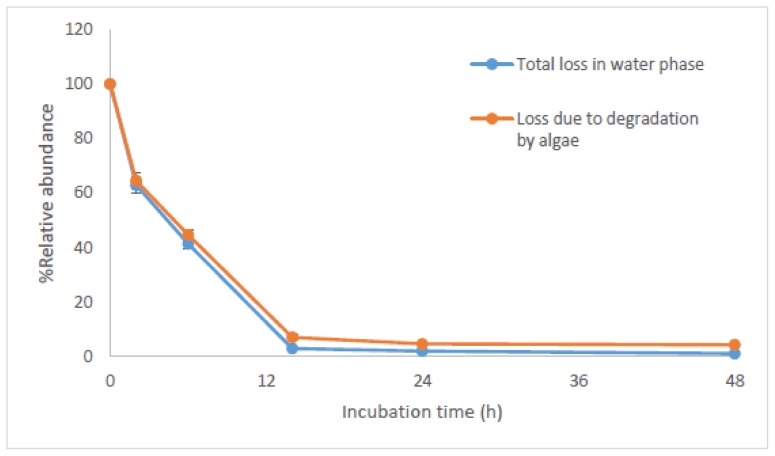
Dissipation of the total NPnEOs (*n* = 12) in the algal (*C. vulgaris*) culture water.

To clarify the dissipation pathways of NPnEOs, the loss due to degradation were calculated based on mass balance (Figure 6). It can be found that the removal rate of NPnEOs (*n* = 1–12) in water was very fast in the first 2 h, which became relatively slower after 2 h. The concentration of the total NPnEOs (*n* = 1–12) reduced to 0.21 μg/L at 14 h, which is 3.01% of the initial concentration, which means 97.0% of the initial 16.94 mg/L NPnEO was removed from water. After 14 h, the concentration of NPnEOs (*n* = 1–12) remained relatively constant, and the removal ratio reached 98.9% at the end of the experiment.

Degradation followed similar kinetic trend with the total dissipation. The degradation rate of NPnEOs (*n* = 1–12) by *C. vulgaris* was fast in the first 14 h, reaching a maximum rate of 3.84 mg/h at 2 h and falling to 1.04 mg/h in the period of 2–14 h (Figure 6). The degradation efficiency was 92.9% at 14 h, and the concentration of NPnEOs (*n* = 1–12) in water reduced to 0.49 mg/L from the initial concentration (16.94 mg/L). The degradation continued after 14 h, but the degradation rate was so slow that the degradation efficiency just increased 2.8%–95.7%. It is evident that NPnEOs (*n* = 1–12) degradation is rapid and almost complete within the first 14 h, which has a similar tendency with NPnEOs (*n* = 1–12) degradation using acclimated sludge [64]. The removal rates of all NPnEO homologues (*n* = 1–12) by *C. vulgaris* are listed in Table 2. Apart from NP1EO and NP2EO, the other homologues were degraded by *C. vulgaris* almost by 100% after 48 h experiment. Jonkers *et al.* [62] investigated the kinetics of aerobic biodegradation of NPnEOs (*n* = 4–15) in a laboratory scale bioreactor filled with river water, the concentration of NPnEOs (*n* = 4–15) reduced to 50% after 10 h and over 99% of the initial NPnEOs was degraded after 4 days. The long chain NPnEOs degraded to short chain NPnEOs in aerobic conditions with a relative fast rate in both studies. In this study, NP1EO and NP2EO were accumulated in water phase due to their slow degradation rates (Table 2).

**Figure 7.**
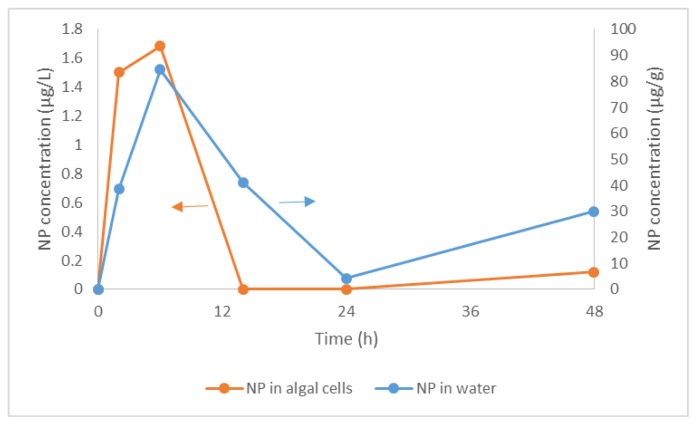
Concentration variation of NP in water and algal cells during the bioconcentration and biodegradation of 16.94 mg/L NPnEO (*n* = 1–12) by *C. vulgaris*.

**Figure 8.**
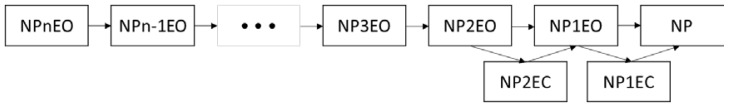
Aerobic biodegradation pathway of NPnEOs [62].

In the experiment, NP was detected as one of the metabolites both in water and algal cells. As shown in Figure 7, the concentration of NP in water and algal cells reached the maximum at 6 h, and then declined to a minimum at 14 h in water and 24 h in the algal cells, followed by a slight elevation. During the first 6 h, the increasing concentration of NP could be due to the degradation of NPnEOs. It has been reported that besides to the consecutive shortening of EO chain (Figure 8), short chain NPnEOs can be oxidized to the corresponding nonylphenoxyethoxy acetic acids (NPnEC) [62], then breakdown to NP. We did detect NPnEC (not quantified) in the experimental system, both breakdown pathways might have been involved in the degradation of NPnEOs. It is the involvement of NPnEC that leads to the similar complicated change tendency of NP, NP1EO and NP2EO, especially in algae cells, which first increased and then decreased and increased again. The second elevation is thought to be the result of degradation of NPnEC.

BCF of NP was about 50,000 at 6 h and 252,000 at 48 h. These values are much higher than the data above when NP was spiked alone and in the literature, which suggests that degradation of NPnEOs occurred inside the algal cells. Hence, as a metabolite of degradation, NP accumulated in the algae, and steady state of bioconcentration between the algae and water had not been achieved.

## 3. Experimental Section

### 3.1. Chemicals and Algae

The standard of NP and NPnEOs (*n* = 1–15) were purchased from Tokyo Chemical Industry (Tokyo, Japan) and Hayashi Pure Chemical (Osaka, Japan), respectively, with the purity of 99.9%. An industrial product was purified by collecting HPLC elute of NPnEOs (*n* = 1–12) and used in bioconcentration and degradation test, and its concentration and homologue distribution was determined. The freshwater green algae, *Chlorella vulgaris*, were bought from the Institute of Hydrobiology, Chinese Academy of Science, Wuhan, China. They were cultured sterilely in Bold Basal medium at 25 ± 1 °C [65]. The light intensity was 50 mmol photons/m^2^/s with a 12:12 light-dark cycle.

### 3.2. Bioaccumulation and Biodegradation of NP and NPnEOs by *C. vulgaris*

The tests were carried out in 3-L Erlenmeyer flasks with concentration fixed at 200 μg/L for NP and 16.94 mg/L for NPnEOs (*n* = 1–12), respectively. The Bold Basal medium was the base solution when the tests were conducted and the algal cells were acclimated in the medium under the same conditions as mentioned above. After arriving at the middle exponential growth phase, the cells were collected, rinsed with the fresh medium and then resuspended into the different test media under sterilization condition, the initial cell density was in the range of 1–2 × 10^5^ cells/mL. The algal culture was shaken every three hours to ensure the exchange of gases. A 500 mL aliquot was taken out at 2, 6, 14, 24 and 48 h, respectively, and divided into two samples for duplicate analysis. After separating algal cells from water by filtration using the 0.45 μm fiber filter, the concentrations of NP and NPnEOs were determined both in algal cells and in culture solution. Control was conducted as the same condition without the introduction of algae.

### 3.3. Sample Pre-Treatment

Water samples were subjected to a concentration and clean-up step by solid-phase extraction (SPE) using Waters Oasis TM HLB cartridges (Milford, MA, USA) [66]. The SPE cartridges were preconditioned with 2.0 mL of DCM/methanol (50/50, *v*/*v*), followed by 1.0 mL of methanol, and then equilibrated with 1.0 mL of super-pure water. The samples were passed through the cartridge at a flow rate of 1 mL/min, and the cartridge was cleaned with 1.0 mL of methanol/water (95/5, *v*/*v*) and eluted with 2.0 mL of DCM/methanol (50/50, *v*/*v*). The eluent was evaporated to almost dryness at 0.008 MPa, 50–52 °C in a water bath, and 1.0 mL of *n*-hexane was then added to redissolve the residue before HPLC analysis.

Liquid-liquid extraction was used to extract the NP and NPnEOs (*n* = 1–12) in algal cells. The algal cells were suspended in 8 mL of water, and crushed by ultrasonic cell-crushed-apparatus for 2 min after being freezed-thawed for 4 cycles in the darkness. The crushed algal cells were extracted twice using 4-mL dichloromethane, and the organic phase was combined and evaporated to almost dryness by rotary evaporation. The residue was redissolved in 1.0 mL of *n*-hexane, and the solution was filtrated using 0.45 μm organic film prior to HPLC (High Performance Liquid Chromatography) analysis.

### 3.4. HPLC Analysis

The HPLC analysis was performed using a Waters 1552 high performance liquid chromatograph equipped with a fluorescence detector and a Millennium software package [66]. A μBondapak Amino (NH_2_) Column (Waters-125Å, Milford, MA, USA, 10 μm, 3.9 mm × 300 mm, 1/pkg) was employed under ambient temperature. Mobile phase flew at a flow rate of 1.0 mL/min and the injection volume was 20 μL. Separation was performed with a linear gradient elution of solvent A (*n*-hexane/isopropanol (98/2, *v*/*v*)) and solvent B (isopropanol/water, (98/2, *v*/*v*)) (Table 3). The fluorescence detection was achieved at an excitation wavelength of 233 nm and an emission wavelength of 302 nm. The analytical method can simultaneously quantify NPnEOs (*n* = 1–12) and their small inter-metabolites in samples. The detection limits of the method are 0.01 μg/L for NP and NP1EO, 0.02 μg/L for NP2EO and NP3EO, 0.05 μg/L for NP4EO and the following long chain homologues, respectively. Recoveries for different NPnEOs and NP were larger than 90% in water phase and 88% in algae.

## 4. Conclusions

Bioconcentration and degradation of NP and NPnEOs (*n* = 1–12) by a pure culture of *C. vulgaris* were studied for the first time. NP and NPnEOs (*n* = 1–12) can be concentrated by *C. vulgaris*, and the 48 h BCFs were 2422 and 13,831, respectively. BCF increased as the EO chain became shorter. Biodegradation of both NP and NPnEOs did occur, which accounted for 56.7% of NP loss and 98.7% of NPnEOs loss in water, respectively. The high BCF value of NPnEOs compared to NP is thought to be due to the biodegradation, which is thought to occur in algal cells and generate NP and short chain NPnEOs, such as NP1EO and NP2EO. The degradation products of NPnEOs were mainly NP, NP1EO and NP2EO, and the content of NP1EO was the largest at the end of the 48 h experiment. Though not quantified, NPnEC was detected, and the degradation pathway through NPnEC is thought to account for the complicate change in concentration of NP1EO, NP2EO and NP, especially in algal cells.

As primary producers of aquatic ecosystem, microalgae can concentrate a mass of NP and NPnEOs (*n* = 1–12) which can be transferred to organisms at higher trophic levels through the food chain, such as invertebrate Daphnia magna. In addition, the degradation products—for instance, short chain NPnEO homologues and NP—exhibit greater toxicity than the parent compounds. Thus, this work shows that the environmental fate of NP and NPnEOs concentrated by microalgae should be considered as a risk for the organisms living in the same ecosystem.

## Figures and Tables

**Table 1.** BCFs of NPnEO homologues (*n* = 1–10) by *C. vulgaris* at different times.

Homologue	BCF at different time (h)				

	2	6	14	24	48
NP1EO	564	1186	1857	2271	11,028
NP2EO	174	345	22,350	7500	10,294
NP3EO	103	176	3727	3571	6750
NP4EO	75	682	2950	1821	4000
NP5EO	343	2783	2204	3357	7065
NP6EO	49	72	1200	1100	2416
NP7EO	32	71	725	585	1270
NP8EO	27	65	579	485	874
NP9EO	19	61	204	232	459
NP10EO	15	48	86	136	171
Total NPnEOs	155	448	6563	5406	13,831

**Table 2.** Removal rates of all NPnEO homologues (*n* = 1–12) by *C. vulgaris* in 48 h.

Homologue	Removal rate of NPnEO homologue in water phase (%)	Degradation rate of NPnEO homologue in algal culture (%)
NP1EO	−1356 *	-
NP2EO	−1.59 *	-
NP3EO	89.6	88.0
NP4EO	96.9	97.7
NP5EO	99.9	88.7
NP6EO	99.1	98.6
NP7EO	99.4	99.0
NP8EO	99.3	98.8
NP9EO	99.3	98.8
NP10EO	99.1	98.6
NP11EO	98.8	98.7
NP12EO	100	100

* The negative sign means increase in mass.

**Table 3.** The linear elution gradient for HPLC analysis of NPnEOs and their small metabolites.

Time (min)	*n*-hexane/isopropanol (98/2, *v*/*v*)	isopropanol/water (98/2, *v*/*v*)
0	5	95
30	50	50
35	70	30
40	65	35
45	50	50
55	5	95
